# The cognitive status of chronic subdural hematoma patients after treatment: an exploratory study

**DOI:** 10.1007/s00701-023-05508-7

**Published:** 2023-02-08

**Authors:** Jurre Blaauw, Heleen M.den Hertog, Dana C. Holl, Nikki S. Thüss, Niels A. van der Gaag, Korné Jellema, Ruben Dammers, Kuan H. Kho, Rob J. M. Groen, Hester F. Lingsma, Bram Jacobs, Joukje van der Naalt

**Affiliations:** 1 Department of Neurology, University of Groningen, University Medical Center Groningen, Groningen, Netherlands; 2 Center for Medical Decision Sciences, Department of Public Health, Erasmus Medical Center, Rotterdam, Netherlands; 3 Department of Neurology, Isala Hospital Zwolle, Zwolle, Netherlands; 4 Department of Neurosurgery, Erasmus MC Stroke Center, Erasmus Medical Center, Rotterdam, Netherlands; 5 Department of Neurology, Subdepartment of Neuropsychology, University of Groningen, University Medical Center Groningen, Groningen, Netherlands; 6 Haaglanden Medical Center &, University Neurosurgical Center Holland (UNCH), Leiden University Medical Center, Haga Teaching Hospital, Leiden & The Hague, Netherlands; 7 Department of Neurology, Haaglanden Medical Centre, The Hague, Netherlands; 8 Department of Neurosurgery, Medisch Spectrum Twente, NeurocenterEnschede, Netherlands; 9 Clinical Neurophysiology Group, Faculty of Science and Technology, University of Twente, Enschede, Netherlands; 10 Department of Neurosurgery, University Medical Center Groningen, Groningen, Netherlands

**Keywords:** Chronic subdural hematoma, Cognitive status, TICS-m, Cognition

## Abstract

**Objective:**

Chronic subdural hematoma (CSDH) is a common neurological condition, often affecting the elderly. Cognitive impairment is frequently observed at presentation. However, the course and longer term aspects of the cognitive status of CSDH patients are unknown. In this study, we aim to explore the cognitive status of CSDH patients after treatment.

**Methods:**

An exploratory study in which CSDH patients were assessed 3 months after treatment and compared to healthy controls. A total of 56 CSDH patients (age 72.1 SD ± 10.8 years with 43 [77%] males) and 60 healthy controls were included (age 67.5 ± SD 4.8 with 34 [57%] males). Cognitive testing was performed using the Telephonic Interview of Cognitive Status-modified (TICS-m), a 12-item questionnaire in which a total of 50 points can be obtained on several cognitive domains.

**Results:**

Median time between treatment and cognitive testing was 93 days (range 76–139). TICS-m scores of CSDH patients were significantly lower than healthy controls, after adjusting for age and sex: mean score 34.6 (95% CI: 33.6–35.9) vs. 39.6 (95% CI: 38.5–40.7), *p value* < *0.001*. More than half (54%) of CSDH patients have cognitive scores at follow-up that correspond with cognitive impairment.

**Conclusion:**

A large number of CSDH patients show significantly worse cognitive status 3 months after treatment compared to healthy controls. This finding underlines the importance of increased awareness for impaired cognition after CSDH. Further research on this topic is warranted.

## Introduction

Chronic subdural hematoma (CSDH) is one of the most common neurological diseases, especially prevalent in elderly patients [19]. The incidence of CSDH is reported to be 17.6/100,000/year in patients below 70 years of age, increasing to 129.5/100,000/year in those aged 80 and over [29]. This incidence is expected to rise even further as a result of the ongoing ageing of the population [11].

Patients with CSDH patients can have various symptoms including headache, (progressive) hemiparesis, gait disturbances, and cognitive complaints [19]. Symptom prevalence may vary with age as younger patients more often show signs of increased intracranial pressure (headache, nausea, vomiting), whereas cognitive impairment is more frequent in elderly patients [1, 12, 35]. Furthermore, in a recent meta-analysis, we have shown that the overall incidence of impaired cognitive status as presenting symptom is high (45%) in CSDH patients regardless of their age [3]. Other studies found that cognitive impairment improves after treatment, and even named it a “reversible cause of dementia” [13, 14, 16, 26, 30]. However, these studies all had relatively short intervals between treatment and testing, which varied between 24 h and 2 weeks. As a result, the cognitive status of CSDH patients on the longer term is not known. Taking the overall incidence of cognitive impairment in elderly into account, comparison to a group of non-CSDH patients is warranted before statements about the effect of CSDH on cognition can be made [22].

Cognitive complaints are known to have a large influence on the ability to live independently and on quality of life [9]. Also, it is known that specific training programs can result in improved cognition and outcome [9, 10, 23]. Therefore, analyzing cognition of CSDH patients might facilitate better (cognitive) outcomes, by allowing the development of specific training programs.

The aim of our study was to prospectively assess the cognitive status of CSDH patients in comparison with a cohort of elderly healthy controls and study the association of cognitive status with the management of CSDH.

## Methods

### Study population

We included 56 CSDH patients who were regarded eligible for a multicenter randomized controlled trial comparing intervention options for CSDH, out of 104 screened patients [24]. Mean age was 72.1(± 10.8) years and 43 (77%) were male. For patients characteristics and clinical background, see Table 1.

**Table 1:** Demographics and clinical background of included CSDH patients

Variable	CSDH patients *n* = 56
Age, years (± SD)	72.1 (± 10.8)
Male sex, *n* (%)	43 (77)
Markwalder grading scale baseline, *n* (%)	
- MGS 0	1 (2)
- MGS 1	41 (73)
- MGS 2	14 (25)
- MGS 3	0 (0)
- MGS 4	0 (0)
Altered mental status at presentation	20 (36)
Side hematoma, *n* (%)	
- Left	23 (41)
- Right	21 (38)
- Bilateral	12 (21)
Treatment modality, *n* (%)	
- Surgery	32 (57)
- Surgery + dexamethasone	18 (32)
- Dexamethasone	4 (7)
- Other (start of anti-epileptic drugs)	2 (4)
Diagnosis to follow-up, days (median, range)	95 (84–183)
Start treatment to follow-up, days (median, range)	93 (76–139)
Surgery to follow-up, days (median, range)	90 (42–103
Dexamethasone to follow-up, days (median, range)	95 (67–130)
Glasgow Outcome Scale-Extend (GOS-E) score at follow-up, *n* (%)	
- Upper good recovery (8)	17 (30)
- Lower good recovery (7)	15 (27)
- Upper moderate disability (6)	15 (27)
- Lower moderate disability (5)	3 (5)
- Upper severe disability (4)	5 (9)
- Death (1), vegetative state (2), lower severe disability (3)	0 (0)
- Unknown	1 (2)

Out of 56 CSDH patients, the MGS score 1 was present in 41 patients (73%). Hematomas were almost evenly distributed between left (23 patients (43%)) and right (21 patients (38%)) side, and 12 patients (21%) had a bilateral CSDH. The majority (50 patients, 89%) received surgical treatment, of which 32 received solely surgery (64%) and 18 additional dexamethasone (36%). Median time from treatment to TICS-m administration was 93 days (range 76–139) (see Table 1 for patients characteristics and clinical background).

The current study was done in three participating centers. Only patients with Markwalder grading scale (MGS; for explanation, see below) scores 0–2 were included to prevent interaction with severity of consciousness that could hamper the assessment of the cognitive status.

In short, inclusion criteria for this study on cognitive status comprised symptomatic CSDH, with age above 18 years. We excluded patients with a history of cerebrospinal fluid shunts, cerebral tumors, and medical history deemed to influence cognitive status (e.g., advanced multiple sclerosis, diagnosed or suspected dementia, severe cerebral infarction, or intracranial hemorrhage). Eligible CSDH patients were screened between July 2020 and May 2021.

All CSDH were diagnosed by certified (neuro)radiologists with routine head CT and radiologically defined as a crescent hematoma with a maximum of one-third hyperdense components in the hematoma. The used treatment modalities in the three neurosurgical centers were overall similar. Surgery was performed through burr hole craniostomy or craniotomy at the discretion of the treating physician. Postoperative drainage was routinely performed with a subdural drain, unless this was technically impossible (e.g., as a result of small hematoma volume.) Dexamethasone was administered in a tapering course of 19 days starting with two times 8 mg daily.

Data were collected on age, sex, medical history, and presence of cognitive complaints at baseline. The definition of cognitive complaints was based on the findings of our systematic review regarding cognitive symptoms in CSDH patients [3] and was defined as the presence of memory deficits, slowed cognitive functioning (bradyphrenia), disorientation, and/or altered behavior, either self-reported or established by the treating physician. All included patients were actively asked for any of the above listed signs/symptoms at presentation. Cognitive complaints at baseline were regarded present when patients fulfilled one or more criteria.

Clinical severity of CSDH was assessed with the Markwalder grading scale (MGS), a 5-point grading scale specifically designed for CSDH ranging from 0 neurologically normal to 4 comatose [21]. Also the laterality of CSDH and treatment modality (surgery, dexamethasone, surgery + dexamethasone) were registered. We collected date of diagnosis, treatment, and follow-up and calculated time intervals between these dates. At 3 months post treatment, we assessed functional status using the Glasgow Outcome Scale-Extended (GOS-E) score, a score varying from 1 to 8 in which 1 is death and 8 is full recovery [34]. This study was approved by the medical ethical committee “Leiden-Den Haag-Delft,” and local approval was obtained from the participating centers. Informed consent was obtained in all participants.

### Control group

The TICS-m was administered in a group (*n* = 60) of healthy controls aged 60 years and older. Mean age was 67.5 (± SD 4.8) years and 34 (57%) were male. The control group was recruited using information leaflets and posters that were distributed in the social and working environment of the researchers.

These controls were considered healthy if they had no medical history of cerebral infarction, bleeding or tumor, no dementia or analysis for (suspected) dementia, no history of (hospitalization for) traumatic brain injury, and no use of drugs that might impair cognition (e.g., valproic acid). Age at time of TICS-m administration and sex were collected for the healthy controls. The medical ethical committee of the University Medical Center Groningen considered this study not to fall within the scope of Dutch Medical Research Involving Human Subjects Act (WMO). Informed consent was obtained for all healthy controls.

Healthy controls were significantly younger (*p value* < *0.001*) and more often female than the CSDH patients *(p value 0.022).*

### Cognitive status

Cognitive status was assessed at three months post treatment, using the Dutch version of the Modified Telephone Interview for Cognitive Status (TICS-m) [17, 33]. The TICS-m contains 12 items for which points are given, leading to a maximum score of 50 points (see Table 2)*.* A cut-off ≤ 34 is set to indicate cognitive impairment, and a score of ≤ 28 indicates dementia [7, 8]. The TICS-m addresses aspects of orientation, attention, and repetitive calculation and includes a 10-item word list with direct and delayed recall.

**Table 2:** Items of the Modified Telephone Interview for Cognitive Status (TICS-m) with corresponding maximum number of points and classification by cognitive domain. The twelve items are listed in the order in which the TICS-m is administered

TICS-m item		Points	Suggested cognitive domain
1	Full name	2	-
2	Orientation (“date, day of week, season”)	5	*Orientation/mental tracking*
3	Age and phone number	2	*-*
4	Counting backwards from 20 to 1	2	*Orientation/mental tracking*
5	Ten word list direct recall	10	*Verbal memory*
6	Count backward from 100 by 7 s *(sequential calculation)*	5	*Attention/working memory*
7	Naming (“tool to cut paper?”)	4	*Language/reasoning*
8	Repeat phrases (“methodist episcopal”)	2	*Attention/working memory*
9	Topical information (“name of prime minister and king”)	4	*-*
10	Tap five times on phone	2	*Orientation/mental tracking*
11	Opposites (“opposite of east”)	2	*Language/reasoning*
12	Ten word list delayed recall	10	*Verbal memory*

The TICS-m was administered by two of the authors (JB and NST), who are skilled neuroscientist with much experience in the assessment of the TICS-m. The TICS-m has several similarities with the Modified Mini-Mental State Exam (3MS), with the advantage of administration by phone. Most items of the TICS-m have been demonstrated to correspond with different cognitive domains: orientation/mental tracking, verbal memory, language/reasoning, and attention/working memory (for specific subdivision, see Table 1) [7, 32].

### Statistical analysis

Age, sex, and scores on individual items and subdivision on cognitive domains of the TICS-m score were compared between the CSDH group and the healthy controls, using chi-square, unpaired *t* test or Mann–Whitney *U* test depending on variable type and distribution. The separate items, cognitive domains, and total TICS-m scores of the groups were compared after adjusting for age and sex using ANCOVA analysis.

Using one-way ANOVA, the differences in mean TICS-m score for the variables sex, baseline MGS score, presence of cognitive complaints at baseline, treatment modality, side of hematoma, and GOS-E score at follow-up were tested. When significant differences were present, variables were adjusted for age and sex with ANCOVA analysis.

## Results

### Cognitive status

Of the 56 included CSDH patients, 30 (54%) had a TCIS-m score lower than 35, 3 months after treatment. Out of the 60 healthy controls, seven (12%) had a TICS-m score lower than 35. Six (11%) CSDH patients had a TICS-m score of ≤ 28, and zero healthy controls had a score of ≤ 28.

Following this, CSDH patients had a significantly lower total TICS-m score (mean score 34.3 ± SD 4.8 vs 40.1 ± SD 4.3, *p value* < *0.001*) compared to healthy controls and lower scores on the items “counting backwards,” “ten-word direct recall,” “serial 7,” and “ten word delayed recall” (for scores, see Table 3)*.* Other items of the TICS-m did not differ between the groups. After adjusting for age and sex, total TICS-m score was still lower in CSDH patients than the control group (mean score 34.6 [95% CI: 33.6–35.9] vs. 39.6 [95% CI: 38.5–40.7], *p value* < *0.001*) (see Table 3)*.*

**Table 3:** Modified Telephone Interview for Cognitive Status (TICS-m) scores of CSDH patients vs. healthy controls. Adjustments made for age and sex using one-way ANCOVA. 95% confidence intervals (CIs) rounded at 1 decimal

TICS-m score item (maximum number of points)	Mean score CSDH patients ± SD (*n* = 56)	Mean score healthy controls ± SD (*n* = 60)	*p value*	Adjusted mean score CSDH patients (95% CI) (*n* = 56)	Adjusted mean score healthy controls (95% CI) (*n* = 60)	*p value*
Name (2)	2.0 ± 0.0	2.0 ± 0.0	*1.000*	2.0 (2.0–2.0)	2.0 (2.0–2.0)	*1.000*
Orientation ( 5)	4.7 ± 0.5	4.9 ± 0.3	*0.117*	4.7 (5.7–4.9)	4.7 (4.8–5.0)	*0.124*
Age/phone number. (2)	1.9 ± 0.4	1.9 ± 0.3	*0.924*	1.9 (1.8–1.9)	1.9 (1.8–1.9)	*0.853*
Counting backwards (2)	1.9 ± 0.3	2.0 ± 0	*0.036*	1.9 (1.8–1.9)	1.9 (1.9–2.0)	*0.054*
Immediate recall ten word list(10)	3.9 ± 1.7	6.5 ± 1.7	< *0.001*	4.2 (3.8–4.7)	6.2 (5.8–6.6)	< *0.001*
Sequential calculation (5)	3.9 ± 1.5	4.5 ± 0.9	*0.022*	3.8 (3.5–4.2)	4.6 (4.2–4.9)	*0.003*
Naming (2)	3.7 ± 0.5	3.8 ± 0.4	*0.057*	3.7 (3.6–3.8)	3.9 (3.7–4.0)	*0.020*
Repeat phrase (2)	1.7 ± 0.5	1.8 ± 0.4	*0.164*	1.7 (1.6–1.8)	1.8 (1.7–1.9)	*0.301*
Topical information (4)	3.7 ± 0.7	3.7 ± 0.8	*0.068*	3.8 (3.6–3.8)	3.5 (3.4–3.8)	*0.089*
Tap five times on phone (2)	1.8 ± 0.4	1.9 ± 0.2	*0.116*	1.9 (1.8–1.9)	1.9 (1.9–2.0)	*0.247*
Opposites (2)	1.8 ± 0.3	1.9 ± 0.2	*0.066*	1.9 (1.8–1.9)	1.9 (1.9–2.0)	*0.047*
Delayed recall ten word list (10)	2.9 ± 1.7	5.0 ± 1.9	< *0.001*	3.1 (2.6–3.6)	4.9(4.4–5.3)	< *0.001*
Total score (50)	34.3 ± 4.8	40.1 ± 4.3	< *0.001*	34.6 (33.6–35.9)	39.6 (38.5–40.7)	< *0.001*
*Cognitive domains (maximum number of points)*						
Orientation/mental tracking (9)	8.5 ± 0.7	8.8 ± 0.3	*0.009*	8.6 (8.4–8.7)	8.9 (8.7–9.0)	*0.015*
Verbal memory (20)	6.9 ± 3.3	11.5 ± 3.5	< *0.001*	7.3 (6.5–8.2)	11.0 (10.2–11.9)	< *0.001*
Language/reasoning (6)	5.6 ± 0.7	5.8 ± 0.4	*0.015*	5.6 (5.4–5.7)	5.8 (5.7–6.0)	*0.006*
Attention/working memory (7)	3.6 ± 0.6	3.8 ± 0.3	*0.028*	3.6 (3.5–3.8)	3.8 (3.7–3.9)	*0.079*

In further analyses, patients with cognitive complaints at baseline had a significantly lower TICS-m score than those without (mean score 32.1 ± SD 4.3 vs. 35.5 ± SD 4.5, *p value 0.010*). Also, patients with an MGS score of 2 had lower TICS-m scores than patients with an MGS score < 2 (mean score MGS 2, 31.5 ± SD 4.4 vs. MGS < 2, 35.2 ± SD 4.5, *p value* < *0.001) *(see Table 4)*.*

**Table 4:** Modified Telephone Interview for Cognitive Status Modified (TICS- m) scores for patients with and without cognitive complaints at baseline and patients with Markwalder grading scale (MGS) score of 2 and < 2 at baseline

	Patients with cognitive complaints	Patients without cognitive complaints	*p* value
Mean TICS-m score ± SD	32.1 ± 4.3	35.5 ± SD 4.5	*0.010*
Adjusted mean TICS-m score (95% CI)*	32.0 (30.0–33.9)	35.6 (34.1–37.0)	*0.004*
Patients with MGS < 2		Patients with MGS 2	
Mean TICs-m score ± SD	35.2 ± 2	31.5 ± 4.4	< *0.001*
Adjusted mean TICS-m score (95% CI)*	35.1 (33.7–36.5)	32.0 ( 29.5–34.4)	0.095

^*^Adjusted for age and sex

No statistical differences were found for treatment modality, side of hematoma or GOS-E score at follow-up*.* After adjusting for age and sex, patients with cognitive complaints (mean score 32.0 [95% CI: 30.0–33.9] vs. 35.6 [95% CI: 34.1–37.0), *p value 0.004*) had significantly lower scores. No differences were found when comparing the TICS-m for the MGS 2 and < 2 scores at baseline after adjustment (mean score MGS 2, 32.0 [95% CI: 29.5–34.4] vs. MGS < 2, 35.1 [95% CI: 33.7–36.5], *p value 0.095*).

### Cognitive subdomains

When comparing the four cognitive domains, CSDH patients scored significantly worse on all domains, compared to the healthy controls. After adjustment for age and sex, CSDH patients scored lower on three of the four cognitive domains: “orientation/mental tracking,” “verbal memory,” and “language/reasoning.”

## Discussion

In this exploratory study, we have found that CSDH patients have significantly worse cognitive status 3 months after treatment compared to healthy controls as detected with a telephonic screening test, the TICS-m. More than half of CSHD patients suffer from objective cognitive deficits in the subacute phase after treatment with a TICS-m score of less than 35 points at follow-up. These deficits were not related to severity of CSDH on admission or treatment modality.

The mean scores on the TICS of 34.3 were significantly lower in patients compared to healthy controls. When comparing the TICS-m scores of our CSDH patients to patients’ scores of other studies, CSDH patients are found to score higher than patients with dementia, mild cognitive impairment (MCI), and schizophrenia [7, 18, 20]. Especially the differences with MCI are interesting. The patients with MCI in one of these studies [7] were older (median age 81) than our CSDH patients, and their TICS-m scores were lower (median 29 [IQR 26–32] vs. mean 34). This might indicate that although a CSDH does affect the cognitive status of CSDH patients, it results in less severe impairment than in patients with MCI and might include scores within the spectrum of healthy controls. Another recent study, investigating patients with ischemic stroke, presented more similar scores to our cohort, with 34% of their patients having a TICS-m score of < 32 [4]. The chosen cut-off point of 32 for cognitive impairment in their study underscores the variety in chosen cut-off points as many other studies set a score of < 34 for mild cognitive impairment and < 28 for dementia [8]. Therefore, we believe that comparing (and adjusting for confounders) mean scores amongst patients and healthy controls, as we did in our study, provides more detailed information than dichotomization.

Regarding the time interval between testing and diagnosis of CSDH, we measured cognitive status in the subacute phase after treatment at 3 months, whereas a similar study in CSDH assessed the cognitive status at an even longer interval of 5.5 years after diagnosis [25]. Using the Cognitive Telephone Screening Instrument (COGTEL), the cognitive status of 51 CSDH patients was compared to a group of healthy controls. Although the COGTEL is an instrument that is less frequently applied [5], their results are interesting as CSDH patients had similar overall scores on the COGTEL compared to healthy controls, with only lower scores on short-term verbal memory. Although this finding differs from our results, this aforementioned testing interval might indicate an improvement of cognitive status over time and not reflecting eventual deterioration due to ageing. However, for this assumption, further research is needed using more comparable testing methods at different time points.

Before statements can be made about the cognitive status of patients with CSDH, comparison with a healthy control group is necessary and the validity of this group should be discussed. The overall mean TICS-m score in our healthy controls seemed to be low with 39 points out of a possible total of 50. However, when comparing our scores to previous studies, even lower scores are reported. A Korean study that assessed the validity of the TICS-m, reported a mean score of 31.4 in healthy controls [31]. This control group was established after MRI imaging of the brain to exclude any lesions and contained a lower mean education level (± 7 years) compared to an average education level of 12 years present in the Netherlands, explaining this lower score [28]. An American study also reported a lower median score of 34 (IQR: 32–37) [18] and comprised more elderly with median age of 81 as explanation for these lower scores. A study in a Dutch cohort of type 2 diabetes patients and healthy controls reported a comparable mean score of 36.5 [32] with corresponding exclusion criteria. Overall, it seems that our healthy controls scores are comparable to other control groups, when taking age differences, sample size, and comorbidity into account, validating the findings of the decreased TICS-m scores in patients with CSDH.

Another aspect of cognition that we tried to capture was an eventual subdivision in cognitive domains. Especially verbal memory is affected in CSDH patients compared with healthy controls. Scores on the “attention/working memory” domain of the TICS-m did not differ between CSDH patients and healthy controls, after adjustment. The latter suggests that this domain might not be affected by CSDH, but it could also represent a limitation of the TICS-m as the two items that measure verbal recall comprise 20 of the total 50 (40%) points, showing that the TICS-m is predominantly focused on verbal aspects of cognition. Given this finding and the fact that visuo-spatial items are not incorporated in the TICS, underlines the awareness that it has to be regarded as a screening test.

Patients with cognitive complaints at baseline had lower mean TICS-m scores at 3 months than healthy controls. This association of poorer baseline cognitive status with lower postoperative cognitive scores has been described before in CSDH [14, 35]. As these cognitive complaints at admission are only subjective and mostly fall within the spectrum of healthy ageing patients, it is difficult to extrapolate this to clinical practice regarding cognitive outcome.

No statistical differences were found in TICS-m scores for the subgroups of the MGS. This might be explained by the fact that the MGS is not designed to assess cognition as it is a clinical severity scale. The MGS does not incorporate any form of cognitive deficit, except that patient who are classified as MGS 2 are by definition regarded disoriented. When the treatment modality was taken into account, no differences in TICS-m scores in our cohort were present, suggesting that cognitive status is not influenced by management choices. Our results also did not show differences in TICS-m scores in the various categories of the GOS-E. Given the reported effect of cognition on functional outcome, this is a somewhat surprising result. An explanation for this finding concerns the ceiling effect of the GOSE in the upper categories of recovery. The GOS-E is known to measure in particular functional outcome and not cognition. Notably, previous studies have shown that almost half of the patients with a good recovery on the GOS-E still experience cognitive and behavioral problems [2].

### Limitations

Despite the interesting findings, we realize that our explorative study has several limitations. The first limitation is that lack of information on pre-CSDH cognitive functioning of our patients. However, given the significantly lower scores of CSDH patients compared to healthy controls and the fact that we excluded patients with a medical history expected to influence cognitive status (e.g., known dementia), we do not deem it likely that pre-CSDH cognitive impairment alone can account for the lower TICS-m scores that we found. However, since CSDH is linked to cerebral atrophy it cannot be excluded that certain CSDH patients experience to some extent cognitive complaints pre-CSDH already. To account for this, cognitive testing at baseline should be incorporated in future studies. The second limitation of our study is that we compared CSDH patients to a group of (relatively) very healthy controls. Therefore, it might be suggested that we did not measure the effect of CSDH on cognition, but merely the effect of hospitalization. However, given the large difference in scores, we believe that hospitalization alone cannot account for these differences. Also, as we had no information about education of patients or healthy controls it is possible that the differences we found were influenced by socio-economic status. Another limitation is the fact that the TICS-m is a validated tool for screening of cognitive status, and therefore, results should be interpreted with caution. Ideally, we would have liked to compare the TICS-m scores to a comprehensive battery of neuropsychological tests. A telephonic assessment is easy applicable for which no travelling is necessary and a such can be used as a screening test. A limitation of telephonic administration, especially in the elderly, is difficulties with hearing. This could be disadvantageous specifically for the test that used the recall of spoken words.

The difference in age between the CSDH group and our healthy controls poses another limitation, as our control group was about 5 years younger. Statistical correction for age, as we have performed, minimizes this chance of bias.

Finally, our study might comprise a selection bias resulting from the study design comprising a RCT with strict inclusion and exclusion criteria. However, the inclusion of a large number of patients also from the registry (comprising excluded patients) might reduce this bias to some extent. In conclusion, the results of this exploratory study, also given the limited number of included patients, should be interpreted with carefully. Nevertheless, the results of our study suggest that CSDH has some effect on cognitive functioning in the subacute phase after treatment. Further research focusing on the long-term evaluation of cognition in CSDH patients with prospective studies comprising comprehensive cognitive tests seems therefore warranted.

### Conclusion

In this exploratory study, patients with CSDH showed a significantly worse cognitive status 3 months after treatment when compared to healthy controls. More than half of the CSDH patients (54%) show impaired cognition. The application of comprehensive neuropsychological tests in these patients will improve the knowledge of cognitive status in CSDH and is the first step to facilitate appropriate aftercare for patients with CSDH.

## Data Availability

The data that support the findings of this study are available from the corresponding author, upon reasonable request.
